# Supplementary material for: A machine learning approach for efficient multi-dimensional integration
Source: Sci Rep. 2021 Sep 23;11:18965. doi: 10.1038/s41598-021-98392-z (PMC8460840; doi:10.1038/s41598-021-98392-z)
Supplement: Supplementary file 1 — Supplementary Information 1. [file 41598_2021_98392_MOESM1_ESM.pdf]

Supplementary Information for
A machine learning approach for efficient multi-dimensional integration

Boram Yoon[1]

[1]CCS-7, Computer, Computational and Statistical Sciences Division,
Los Alamos National Laboratory, Los Alamos, NM 87545, USA
boram@lanl.gov

N≈5000, D=5, $||\mathbf{c}||_1$=1.0

Integrand family		1	2	3	4	5	6
N		5313(27)	5514(13)	5135.3(3.2)	5520(15)	5255.6(7.5)	5869(30)
N_{train}		2623(14)	2732.0(8.9)	3590.5(2.8)	2735.4(8.0)	2546.9(4.8)	1758(12)
N_{crxn}		2739(25)	2845(19)	1545.4(5.0)	2824(14)	2775(16)	4118(50)
$\sigma_I/\|I\|$	VEG	$5(3)\times10^{-3}$	$3.6(1)\times10^{-4}$	$8.8(1)\times10^{-4}$	$3.7(1)\times10^{-4}$	$5.04(8)\times10^{-4}$	$4.7(3)\times10^{-3}$
	MLP	$1.4(8)\times10^{-4}$	$9.2(3)\times10^{-6}$	$1.31(4)\times10^{-4}$	$8.9(2)\times10^{-6}$	$3.7(1)\times10^{-5}$	$4.6(4)\times10^{-3}$
	GBDT	$2(1)\times10^{-3}$	$1.68(3)\times10^{-5}$	$7.7(1)\times10^{-4}$	$1.72(4)\times10^{-5}$	$1.35(2)\times10^{-4}$	$4.8(3)\times10^{-4}$
	GP	$2(1)\times10^{-5}$	$6.6(4)\times10^{-8}$	$2.69(4)\times10^{-6}$	$6.3(3)\times10^{-8}$	$1.83(4)\times10^{-4}$	$2.4(3)\times10^{-2}$
Gain	MLP	37.9(3.4)	41.2(1.5)	6.91(18)	42.2(1.3)	14.06(57)	1.177(65)
	GBDT	5.06(41)	22.10(86)	1.166(34)	21.83(94)	3.77(10)	10.03(51)
	GP	407(29)	6181(319)	329.0(4.1)	6223(258)	2.785(73)	0.261(22)
$N_{>4\sigma}$	GBDT	–	–	–	–	–	1

N≈5000, D=5, $||\mathbf{c}||_1$=3.0

Integrand family		1	2	3	4	5	6
N		5343(34)	5201.9(7.8)	5256.9(4.0)	5209.7(6.5)	5124.1(3.9)	5759(11)
N_{train}		2653(13)	2526.4(3.5)	3716.3(3.4)	2535.2(4.5)	2493.4(1.8)	1742(16)
N_{crxn}		2720(33)	2751.8(9.0)	1568.3(7.9)	2756.3(3.4)	2585(18)	4053(31)
$\sigma_I/\|I\|$	VEG	$1.1(4)\times10^{-2}$	$5.6(1)\times10^{-4}$	$2.04(2)\times10^{-3}$	$5.8(1)\times10^{-4}$	$6.6(1)\times10^{-4}$	$5.1(3)\times10^{-3}$
	MLP	$3(1)\times10^{-4}$	$6.6(2)\times10^{-5}$	$3.9(7)\times10^{-4}$	$7.5(2)\times10^{-5}$	$1.27(5)\times10^{-4}$	$5.1(5)\times10^{-3}$
	GBDT	$6(3)\times10^{-3}$	$1.33(3)\times10^{-4}$	$1.85(3)\times10^{-3}$	$1.50(3)\times10^{-4}$	$4.10(7)\times10^{-4}$	$8.0(2)\times10^{-4}$
	GP	$5(2)\times10^{-5}$	$4.8(3)\times10^{-7}$	$9.6(1)\times10^{-6}$	$5.4(3)\times10^{-7}$	$6.1(1)\times10^{-4}$	$2.6(3)\times10^{-2}$
Gain	MLP	32.7(1.1)	8.73(28)	6.59(31)	7.87(21)	5.50(19)	1.141(66)
	GBDT	2.18(11)	4.33(16)	1.129(36)	3.95(14)	1.647(45)	6.49(42)
	GP	196.8(1.7)	1321(67)	211.46(98)	1151(47)	1.105(30)	0.253(19)
$N_{>4\sigma}$	VEG	–	–	–	–	–	2

N≈5000, D=5, $||\mathbf{c}||_1$=8.0

Integrand family		1	2	3	4	5	6
N		5245.0(6.9)	5074.6(5.9)	5415.2(5.5)	5280(19)	5113.2(8.7)	5669(22)
N_{train}		2604.3(3.7)	2513.1(4.7)	3860.6(5.2)	2567.0(8.1)	2544.3(5.5)	1801(15)
N_{crxn}		2609(20)	2540.9(7.8)	1535.8(7.9)	2700(24)	2582(11)	3883(25)
$\sigma_I/\|I\|$	VEG	$5(2)\times10^{-2}$	$9.1(1)\times10^{-4}$	$4.38(3)\times10^{-3}$	$1.74(6)\times10^{-3}$	$1.03(2)\times10^{-3}$	$6.2(3)\times10^{-3}$
	MLP	$1.3(7)\times10^{-3}$	$2.71(4)\times10^{-4}$	$8(1)\times10^{-4}$	$3.94(8)\times10^{-4}$	$3.5(1)\times10^{-4}$	$8.5(9)\times10^{-3}$
	GBDT	$9(9)\times10^{-1}$	$6.4(1)\times10^{-4}$	$4.5(1)\times10^{-3}$	$8.4(1)\times10^{-4}$	$1.17(2)\times10^{-3}$	$2.07(6)\times10^{-3}$
	GP	$2(1)\times10^{-4}$	$4.8(8)\times10^{-5}$	$3.3(1)\times10^{-5}$	$5.3(3)\times10^{-6}$	$1.98(4)\times10^{-3}$	$3.3(3)\times10^{-2}$
Gain	MLP	35.72(90)	3.381(81)	7.47(46)	4.52(20)	3.09(11)	0.942(72)
	GBDT	1.200(43)	1.471(64)	0.997(33)	2.15(13)	0.901(37)	3.05(17)
	GP	181.32(57)	47.2(7.9)	137.6(4.5)	346.2(9.4)	0.526(11)	0.220(13)
$N_{>4\sigma}$	VEG	–	–	–	–	–	2

Table S1: Number of integrand evaluations (N, N_{train}, and N_{crxn}), precision of the integral ($\sigma_I/|I|$), precision gain, and $N_{>4\sigma}$ of VEGAS (VEG) and ML (MLP, GBDT, and GP) integration algorithms for $N \approx 5000$ and $D = 5$. The results are averaged over 36 random samples. The numbers in the parentheses are the standard deviation of the mean. Here, $N_{>4\sigma}$ is the number of integration results that are more than 4σ away from the true answer out of the 36 samples; only the non-zero values are presented.

N≈5000, D=8, $||\mathbf{c}||_1$=1.0

Integrand family		1	2	3	4	5	6
N		5000(0)	5000(0)	5000(0)	5000(0)	5000(0)	5000(0)
N_{train}		2500(0)	2500(0)	3500(0)	2500(0)	2500(0)	1500(0)
N_{crxn}		2500(0)	2500(0)	1500(0)	2500(0)	2500(0)	3500(0)
$\sigma_I/\|I\|$	VEG	$9(6)\times10^{-3}$	$5.3(1)\times10^{-4}$	$1.42(1)\times10^{-3}$	$5.21(9)\times10^{-4}$	$7.79(8)\times10^{-4}$	$5.5(4)\times10^{-3}$
	MLP	$2(1)\times10^{-4}$	$1.17(4)\times10^{-5}$	$2.47(7)\times10^{-4}$	$1.09(2)\times10^{-5}$	$9.0(2)\times10^{-5}$	$9(1)\times10^{-3}$
	GBDT	$4(2)\times10^{-3}$	$1.78(2)\times10^{-5}$	$1.90(2)\times10^{-3}$	$1.78(2)\times10^{-5}$	$2.03(2)\times10^{-4}$	$6.4(2)\times10^{-4}$
	GP	$5(3)\times10^{-5}$	$6.9(4)\times10^{-8}$	$6.9(1)\times10^{-6}$	$5.9(2)\times10^{-8}$	$2.45(5)\times10^{-4}$	$3.0(4)\times10^{-2}$
Gain	MLP	40.5(4.0)	46.37(86)	5.89(16)	48.03(96)	8.88(29)	0.714(36)
	GBDT	4.53(37)	30.15(95)	0.752(17)	29.19(52)	3.863(76)	8.62(59)
	GP	352(28)	8292(262)	206.8(2.5)	8918(177)	3.25(10)	0.234(16)
$N_{>4\sigma}$	VEG	–	–	–	–	–	1
	GBDT	–	–	–	–	–	1

N≈5000, D=8, $||\mathbf{c}||_1$=3.0

Integrand family		1	2	3	4	5	6
N		5000(0)	5000(0)	5000(0)	5000(0)	5000(0)	5000(0)
N_{train}		2500(0)	2500(0)	3500(0)	2500(0)	2500(0)	1500(0)
N_{crxn}		2500(0)	2500(0)	1500(0)	2500(0)	2500(0)	3500(0)
$\sigma_I/\|I\|$	VEG	$1.5(6)\times10^{-2}$	$8.1(1)\times10^{-4}$	$3.47(4)\times10^{-3}$	$8.10(9)\times10^{-4}$	$9.4(1)\times10^{-4}$	$5.8(4)\times10^{-3}$
	MLP	$3(1)\times10^{-4}$	$9.1(2)\times10^{-5}$	$6.0(1)\times10^{-4}$	$9.2(2)\times10^{-5}$	$2.71(9)\times10^{-4}$	$1.1(1)\times10^{-2}$
	GBDT	$1.2(6)\times10^{-2}$	$1.49(2)\times10^{-4}$	$5.95(8)\times10^{-3}$	$1.58(2)\times10^{-4}$	$6.17(7)\times10^{-4}$	$1.33(4)\times10^{-3}$
	GP	$1.0(4)\times10^{-4}$	$1.1(2)\times10^{-6}$	$2.67(5)\times10^{-5}$	$5.2(1)\times10^{-7}$	$7.6(1)\times10^{-4}$	$3.1(4)\times10^{-2}$
Gain	MLP	30.2(2.1)	9.17(22)	5.83(14)	8.88(19)	3.64(14)	0.603(29)
	GBDT	1.970(74)	5.52(13)	0.592(15)	5.143(86)	1.542(27)	4.32(22)
	GP	140.5(1.6)	1219(100)	130.4(1.2)	1606(46)	1.270(41)	0.231(16)
$N_{>4\sigma}$	VEG	–	–	–	–	–	2

N≈5000, D=8, $||\mathbf{c}||_1$=8.0

Integrand family		1	2	3	4	5	6
N		5000(0)	5000(0)	5000(0)	5000(0)	5000(0)	5000(0)
N_{train}		2500(0)	2500(0)	3500(0)	2500(0)	2500(0)	1500(0)
N_{crxn}		2500(0)	2500(0)	1500(0)	2500(0)	2500(0)	3500(0)
$\sigma_I/\|I\|$	VEG	$3(1)\times10^{-2}$	$1.12(1)\times10^{-3}$	$8.1(1)\times10^{-3}$	$1.34(2)\times10^{-3}$	$1.24(1)\times10^{-3}$	$8.0(3)\times10^{-3}$
	MLP	$1(1)\times10^{-3}$	$4.38(6)\times10^{-4}$	$1.24(5)\times10^{-3}$	$5.85(9)\times10^{-4}$	$8.0(2)\times10^{-4}$	$1.3(1)\times10^{-2}$
	GBDT	$1(1)\times10^{-1}$	$8.0(1)\times10^{-4}$	$1.85(4)\times10^{-2}$	$1.02(1)\times10^{-3}$	$1.77(2)\times10^{-3}$	$3.47(9)\times10^{-3}$
	GP	$5(2)\times10^{-4}$	$1.0(1)\times10^{-4}$	$1.55(9)\times10^{-4}$	$4.3(1)\times10^{-6}$	$2.23(4)\times10^{-3}$	$3.9(4)\times10^{-2}$
Gain	MLP	32.98(82)	2.588(49)	7.03(31)	2.309(45)	1.613(63)	0.741(51)
	GBDT	1.191(27)	1.420(44)	0.450(10)	1.319(33)	0.707(13)	2.276(71)
	GP	106.5(2.8)	20.2(2.3)	57.2(2.5)	319.4(6.5)	0.568(16)	0.252(18)
$N_{>4\sigma}$	VEG	–	–	–	–	–	2
	MLP	–	–	1	–	–	–

Table S2: Number of integrand evaluations, precision of the integral, precision gain, and $N_{>4\sigma}$ for $N \approx 5000$ and $D = 8$. The notations are the same as Table S1.

N≈5000, D=10, $||\mathbf{c}||_1$=1.0

Integrand family		1	2	3	4	5	6
N		5000(0)	5000(0)	5000(0)	5000(0)	5000(0)	5000(0)
N_{train}		2500(0)	2500(0)	3500(0)	2500(0)	2500(0)	1500(0)
N_{crxn}		2500(0)	2500(0)	1500(0)	2500(0)	2500(0)	3500(0)
$\sigma_I/\lvert I\rvert$	VEG	$6(4)\times10^{-3}$	$4.8(1)\times10^{-4}$	$1.60(1)\times10^{-3}$	$4.48(9)\times10^{-4}$	$8.85(8)\times10^{-4}$	$6.2(4)\times10^{-3}$
	MLP	$2(1)\times10^{-4}$	$1.16(4)\times10^{-5}$	$3.2(1)\times10^{-4}$	$1.08(2)\times10^{-5}$	$1.42(4)\times10^{-4}$	$1.2(1)\times10^{-2}$
	GBDT	$4(3)\times10^{-3}$	$1.68(2)\times10^{-5}$	$2.83(3)\times10^{-3}$	$1.66(2)\times10^{-5}$	$2.32(2)\times10^{-4}$	$7.6(4)\times10^{-4}$
	GP	$6(4)\times10^{-5}$	$5.9(4)\times10^{-8}$	$9.0(1)\times10^{-6}$	$5.0(1)\times10^{-8}$	$2.54(5)\times10^{-4}$	$3.1(4)\times10^{-2}$
Gain	MLP	35.3(3.1)	41.41(88)	5.05(15)	41.78(99)	6.48(23)	0.593(31)
	GBDT	4.37(38)	28.57(93)	0.572(12)	26.95(58)	3.825(66)	8.21(42)
	GP	345(29)	8533(250)	178.4(2.2)	8941(189)	3.55(11)	0.245(15)
$N_{>4\sigma}$	VEG	–	–	–	–	–	1

N≈5000, D=10, $||\mathbf{c}||_1$=3.0

Integrand family		1	2	3	4	5	6
N		5000(0)	5000(0)	5000(0)	5000(0)	5000(0)	5000(0)
N_{train}		2500(0)	2500(0)	3500(0)	2500(0)	2500(0)	1500(0)
N_{crxn}		2500(0)	2500(0)	1500(0)	2500(0)	2500(0)	3500(0)
$\sigma_I/\lvert I\rvert$	VEG	$1.3(5)\times10^{-2}$	$8.91(9)\times10^{-4}$	$3.94(5)\times10^{-3}$	$8.83(8)\times10^{-4}$	$1.06(1)\times10^{-3}$	$6.7(5)\times10^{-3}$
	MLP	$3(1)\times10^{-4}$	$9.6(3)\times10^{-5}$	$8.4(2)\times10^{-4}$	$9.5(1)\times10^{-5}$	$4.4(1)\times10^{-4}$	$1.3(1)\times10^{-2}$
	GBDT	$1.0(4)\times10^{-2}$	$1.42(2)\times10^{-4}$	$9.3(1)\times10^{-3}$	$1.47(1)\times10^{-4}$	$7.11(7)\times10^{-4}$	$1.49(6)\times10^{-3}$
	GP	$1.1(5)\times10^{-4}$	$2.0(5)\times10^{-6}$	$4.0(1)\times10^{-5}$	$4.4(1)\times10^{-7}$	$7.7(1)\times10^{-4}$	$3.3(4)\times10^{-2}$
Gain	MLP	25.2(1.8)	9.51(27)	4.81(15)	9.39(20)	2.477(75)	0.573(32)
	GBDT	1.759(57)	6.31(12)	0.4266(88)	6.002(87)	1.511(28)	4.34(23)
	GP	118.7(1.3)	972(106)	98.7(1.4)	2028(56)	1.405(40)	0.247(15)
$N_{>4\sigma}$	VEG	–	–	–	–	–	3

N≈5000, D=10, $||\mathbf{c}||_1$=8.0

Integrand family		1	2	3	4	5	6
N		5000(0)	5000(0)	5000(0)	5000(0)	5000(0)	5000(0)
N_{train}		2500(0)	2500(0)	3500(0)	2500(0)	2500(0)	1500(0)
N_{crxn}		2500(0)	2500(0)	1500(0)	2500(0)	2500(0)	3500(0)
$\sigma_I/\lvert I\rvert$	VEG	$3(1)\times10^{-2}$	$1.20(1)\times10^{-3}$	$9.3(1)\times10^{-3}$	$1.30(1)\times10^{-3}$	$1.36(1)\times10^{-3}$	$8.1(4)\times10^{-3}$
	MLP	$1.6(8)\times10^{-3}$	$5.05(7)\times10^{-4}$	$1.70(8)\times10^{-3}$	$6.2(1)\times10^{-4}$	$1.20(4)\times10^{-3}$	$1.7(2)\times10^{-2}$
	GBDT	$6(4)\times10^{-2}$	$8.2(1)\times10^{-4}$	$3.33(7)\times10^{-2}$	$9.9(1)\times10^{-4}$	$2.03(2)\times10^{-3}$	$3.9(1)\times10^{-3}$
	GP	$8(4)\times10^{-4}$	$1.1(1)\times10^{-4}$	$2.8(1)\times10^{-4}$	$3.45(9)\times10^{-6}$	$2.23(4)\times10^{-3}$	$4.2(5)\times10^{-2}$
Gain	MLP	25.99(72)	2.389(34)	5.87(26)	2.102(38)	1.184(50)	0.595(39)
	GBDT	1.076(20)	1.472(40)	0.2780(81)	1.321(20)	0.676(14)	2.005(72)
	GP	53.6(1.5)	17.4(1.7)	34.6(1.4)	385.8(9.8)	0.622(16)	0.241(17)
$N_{>4\sigma}$	VEG	–	–	–	–	–	3
	MLP	–	–	5	–	–	–
	GBDT	–	–	1	–	–	–

Table S3: Number of integrand evaluations, precision of the integral, precision gain, and $N_{>4\sigma}$ for $N \approx 5000$ and $D = 10$. The notations are the same as Table S1.

N≈10000, D=5, $||\mathbf{c}||_1$=1.0

Integrand family		1	2	3	4	5	6
N		11720(59)	12372(30)	11269.9(5.5)	12387(22)	11712(14)	13173(95)
N_{train}		5746(33)	6060(19)	7851.1(4.4)	6066(18)	5594.3(6.8)	4083(13)
N_{crxn}		6052(19)	6200(11)	3444(28)	6201.4(8.9)	6164.3(9.1)	9181(103)
$\sigma_I/\lvert I\rvert$	VEG	$3(2)\times10^{-3}$	$1.76(3)\times10^{-4}$	$4.47(5)\times10^{-4}$	$1.72(4)\times10^{-4}$	$2.43(3)\times10^{-4}$	$2.7(1)\times10^{-3}$
	MLP	$7(4)\times10^{-5}$	$4.6(1)\times10^{-6}$	$5.9(1)\times10^{-5}$	$4.4(1)\times10^{-6}$	$1.51(5)\times10^{-5}$	$2.0(2)\times10^{-3}$
	GBDT	$1.5(9)\times10^{-3}$	$9.3(1)\times10^{-6}$	$4.20(5)\times10^{-4}$	$9.5(2)\times10^{-6}$	$7.5(1)\times10^{-5}$	$1.91(9)\times10^{-4}$
	GP	$1(1)\times10^{-5}$	$3.4(2)\times10^{-8}$	$1.50(2)\times10^{-6}$	$3.3(1)\times10^{-8}$	$9.7(2)\times10^{-5}$	$1.5(2)\times10^{-2}$
Gain	MLP	41.7(3.9)	39.9(1.3)	7.65(16)	39.2(1.2)	16.67(54)	1.531(75)
	GBDT	4.51(36)	19.22(54)	1.076(27)	18.42(62)	3.288(97)	14.52(66)
	GP	357(24)	5770(311)	298.9(2.9)	5497(235)	2.546(65)	0.243(18)
$N_{>4\sigma}$	–	–	–	–	–	–	–

N≈10000, D=5, $||\mathbf{c}||_1$=3.0

Integrand family		1	2	3	4	5	6
N		11631(59)	11511(21)	11498.7(8.0)	11483(18)	11325.9(9.1)	12751(39)
N_{train}		5743(20)	5533.6(5.5)	8110.2(6.6)	5543.6(6.0)	5493.7(3.5)	3952(25)
N_{crxn}		5997(21)	6008(10)	3335(14)	5986.5(9.4)	5998(23)	8842(60)
$\sigma_I/\lvert I\rvert$	VEG	$5(2)\times10^{-3}$	$2.83(5)\times10^{-4}$	$1.07(1)\times10^{-3}$	$3.04(6)\times10^{-4}$	$3.40(5)\times10^{-4}$	$2.9(1)\times10^{-3}$
	MLP	$1.3(5)\times10^{-4}$	$3.29(6)\times10^{-5}$	$1.8(2)\times10^{-4}$	$3.9(1)\times10^{-5}$	$4.4(1)\times10^{-5}$	$2.6(3)\times10^{-3}$
	GBDT	$4(2)\times10^{-3}$	$7.5(1)\times10^{-5}$	$1.12(2)\times10^{-3}$	$8.3(1)\times10^{-5}$	$2.32(4)\times10^{-4}$	$3.9(1)\times10^{-4}$
	GP	$3(1)\times10^{-5}$	$2.4(1)\times10^{-7}$	$5.39(7)\times10^{-6}$	$3.0(1)\times10^{-7}$	$3.19(6)\times10^{-4}$	$1.6(2)\times10^{-2}$
Gain	MLP	35.43(98)	8.67(18)	6.90(28)	7.76(17)	7.88(25)	1.262(68)
	GBDT	2.05(11)	3.84(14)	0.975(27)	3.70(12)	1.503(54)	7.47(34)
	GP	174.7(1.6)	1241(44)	200.16(92)	1084(44)	1.076(25)	0.234(15)
$N_{>4\sigma}$	–	–	–	–	–	–	–

N≈10000, D=5, $||\mathbf{c}||_1$=8.0

Integrand family		1	2	3	4	5	6
N		11420(11)	11193.7(8.0)	11814(13)	11517(32)	11244(14)	12519(37)
N_{train}		5677.7(5.1)	5531.6(5.8)	8416(11)	5645(15)	5599.8(8.0)	4008(32)
N_{crxn}		5831(37)	5742(43)	3456(18)	5996(35)	5517(22)	8535(53)
$\sigma_I/\lvert I\rvert$	VEG	$2.0(8)\times10^{-2}$	$4.64(7)\times10^{-4}$	$2.35(1)\times10^{-3}$	$8.5(3)\times10^{-4}$	$5.0(1)\times10^{-4}$	$3.7(1)\times10^{-3}$
	MLP	$5(2)\times10^{-4}$	$1.31(2)\times10^{-4}$	$4(1)\times10^{-4}$	$2.13(6)\times10^{-4}$	$1.32(4)\times10^{-4}$	$3.6(5)\times10^{-3}$
	GBDT	$2(1)\times10^{-2}$	$3.49(9)\times10^{-4}$	$2.91(6)\times10^{-3}$	$4.7(1)\times10^{-4}$	$6.8(1)\times10^{-4}$	$1.06(2)\times10^{-3}$
	GP	$1.4(6)\times10^{-4}$	$1.2(2)\times10^{-5}$	$2.0(3)\times10^{-5}$	$3.2(2)\times10^{-6}$	$1.08(2)\times10^{-3}$	$2.3(2)\times10^{-2}$
Gain	MLP	40.6(1.0)	3.564(87)	7.47(41)	4.13(22)	3.96(12)	1.350(80)
	GBDT	0.997(35)	1.379(57)	0.825(24)	1.86(11)	0.770(32)	3.55(16)
	GP	157.38(47)	121(20)	144.3(6.4)	279.4(7.4)	0.4730(90)	0.205(14)
$N_{>4\sigma}$	VEG	–	–	–	–	–	1

Table S4: Number of integrand evaluations, precision of the integral, precision gain, and $N_{>4\sigma}$ for $N \approx 10000$ and $D = 5$. Notations are the same as Table S1.

N≈10000, D=8, $||\mathbf{c}||_1$=1.0

Integrand family		1	2	3	4	5	6
N		10000(0)	10000(0)	10000(0)	10000(0)	10000(0)	10000(0)
N_{train}		5000(0)	5000(0)	7000(0)	5000(0)	5000(0)	3000(0)
N_{crxn}		5000(0)	5000(0)	3000(0)	5000(0)	5000(0)	6948(27)
$\sigma_I/\lvert I\rvert$	VEG	$7(5)\times10^{-3}$	$3.68(8)\times10^{-4}$	$1.00(1)\times10^{-3}$	$3.55(5)\times10^{-4}$	$5.35(5)\times10^{-4}$	$3.7(2)\times10^{-3}$
	MLP	$9(5)\times10^{-5}$	$6.0(1)\times10^{-6}$	$1.16(3)\times10^{-4}$	$5.7(1)\times10^{-6}$	$3.3(1)\times10^{-5}$	$5.0(5)\times10^{-3}$
	GBDT	$2(1)\times10^{-3}$	$1.11(1)\times10^{-5}$	$1.26(2)\times10^{-3}$	$1.11(1)\times10^{-5}$	$1.30(1)\times10^{-4}$	$3.3(1)\times10^{-4}$
	GP	$3(2)\times10^{-5}$	$4.3(2)\times10^{-8}$	$4.70(9)\times10^{-6}$	$3.7(1)\times10^{-8}$	$1.49(3)\times10^{-4}$	$1.9(2)\times10^{-2}$
Gain	MLP	60.6(5.4)	61.4(1.2)	8.80(23)	62.8(1.2)	16.69(51)	0.791(35)
	GBDT	4.84(38)	33.42(90)	0.802(21)	32.06(52)	4.129(89)	10.95(57)
	GP	359(28)	9130(307)	215.1(2.8)	9649(203)	3.650(98)	0.230(14)
$N_{>4\sigma}$	VEG	–	–	–	–	–	1

N≈10000, D=8, $||\mathbf{c}||_1$=3.0

Integrand family		1	2	3	4	5	6
N		10000(0)	10000(0)	10000(0)	10000(0)	10000(0)	10000(0)
N_{train}		5000(0)	5000(0)	7000(0)	5000(0)	5000(0)	3000(0)
N_{crxn}		5000(0)	5000(0)	3000(0)	5000(0)	5000(0)	6914(39)
$\sigma_I/\lvert I\rvert$	VEG	$1.0(4)\times10^{-2}$	$5.56(7)\times10^{-4}$	$2.51(3)\times10^{-3}$	$5.54(6)\times10^{-4}$	$6.53(8)\times10^{-4}$	$3.8(2)\times10^{-3}$
	MLP	$1.6(6)\times10^{-4}$	$4.68(9)\times10^{-5}$	$3.7(4)\times10^{-4}$	$5.00(9)\times10^{-5}$	$1.02(4)\times10^{-4}$	$5.2(4)\times10^{-3}$
	GBDT	$7(3)\times10^{-3}$	$9.2(1)\times10^{-5}$	$4.12(6)\times10^{-3}$	$9.7(1)\times10^{-5}$	$3.98(5)\times10^{-4}$	$7.3(2)\times10^{-4}$
	GP	$6(2)\times10^{-5}$	$4.6(8)\times10^{-7}$	$1.77(3)\times10^{-5}$	$3.3(1)\times10^{-7}$	$4.6(1)\times10^{-4}$	$2.1(2)\times10^{-2}$
Gain	MLP	46.9(2.7)	11.98(22)	7.66(30)	11.21(22)	6.64(23)	0.786(36)
	GBDT	2.104(85)	6.06(15)	0.620(17)	5.715(72)	1.656(39)	5.04(20)
	GP	147.2(1.5)	1592(89)	142.3(1.2)	1698(49)	1.436(38)	0.228(16)
$N_{>4\sigma}$	VEG	–	–	–	–	–	3
	GBDT	–	–	1	–	–	–

N≈10000, D=8, $||\mathbf{c}||_1$=8.0

Integrand family		1	2	3	4	5	6
N		10000(0)	10000(0)	10000(0)	10000(0)	10000(0)	10000(0)
N_{train}		5000(0)	5000(0)	7000(0)	5000(0)	5000(0)	3000(0)
N_{crxn}		5000(0)	5000(0)	3000(0)	5000(0)	5000(0)	7008(38)
$\sigma_I/\lvert I\rvert$	VEG	$2.6(8)\times10^{-2}$	$7.7(1)\times10^{-4}$	$5.97(6)\times10^{-3}$	$8.8(1)\times10^{-4}$	$8.5(1)\times10^{-4}$	$5.7(2)\times10^{-3}$
	MLP	$7(3)\times10^{-4}$	$2.30(3)\times10^{-4}$	$7.5(5)\times10^{-4}$	$3.10(4)\times10^{-4}$	$2.86(8)\times10^{-4}$	$6.0(6)\times10^{-3}$
	GBDT	$3(2)\times10^{-2}$	$5.08(9)\times10^{-4}$	$1.35(2)\times10^{-2}$	$6.47(8)\times10^{-4}$	$1.16(1)\times10^{-3}$	$2.05(5)\times10^{-3}$
	GP	$3(1)\times10^{-4}$	$4.0(6)\times10^{-5}$	$6.3(2)\times10^{-5}$	$2.8(1)\times10^{-6}$	$1.36(3)\times10^{-3}$	$2.7(3)\times10^{-2}$
Gain	MLP	51.3(1.7)	3.383(78)	8.81(44)	2.851(50)	3.075(89)	1.126(68)
	GBDT	1.252(32)	1.546(47)	0.453(10)	1.368(28)	0.741(17)	2.746(84)
	GP	132.3(2.0)	39.7(5.0)	97.2(2.9)	319.5(7.7)	0.638(15)	0.269(20)
$N_{>4\sigma}$	VEG	–	–	–	–	–	1
	MLP	–	1	3	–	–	–

Table S5: Number of integrand evaluations, precision of the integral, precision gain, and $N_{>4\sigma}$ for $N \approx 10000$ and $D = 8$. Notations are the same as Table S1.

N≈10000, D=10, $||\mathbf{c}||_1$=1.0

Integrand family		1	2	3	4	5	6
N		10000(0)	10000(0)	10000(0)	10000(0)	10000(0)	10000(0)
N_{train}		5000(0)	5000(0)	7000(0)	5000(0)	5000(0)	3000(0)
N_{crxn}		5000(0)	5000(0)	3000(0)	5000(0)	5000(0)	7000(0)
$\sigma_I/\lvert I\rvert$	VEG	$5(3)\times10^{-3}$	$3.4(1)\times10^{-4}$	$1.13(1)\times10^{-3}$	$3.21(5)\times10^{-4}$	$5.90(5)\times10^{-4}$	$3.8(3)\times10^{-3}$
	MLP	$9(6)\times10^{-5}$	$5.9(1)\times10^{-6}$	$1.42(4)\times10^{-4}$	$5.6(1)\times10^{-6}$	$4.8(2)\times10^{-5}$	$6(1)\times10^{-3}$
	GBDT	$3(2)\times10^{-3}$	$1.05(1)\times10^{-5}$	$1.91(2)\times10^{-3}$	$1.04(1)\times10^{-5}$	$1.51(1)\times10^{-4}$	$3.7(1)\times10^{-4}$
	GP	$4(3)\times10^{-5}$	$3.9(2)\times10^{-8}$	$6.3(1)\times10^{-6}$	$3.40(8)\times10^{-8}$	$1.59(3)\times10^{-4}$	$2.1(2)\times10^{-2}$
Gain	MLP	59.3(5.0)	57.6(1.1)	8.16(21)	57.39(95)	12.95(51)	0.696(33)
	GBDT	4.52(40)	32.5(1.1)	0.598(13)	30.96(53)	3.918(71)	9.84(58)
	GP	339(27)	9092(218)	180.5(2.0)	9535(118)	3.78(10)	0.218(11)
$N_{>4\sigma}$	–	–	–	–	–	–	–

N≈10000, D=10, $||\mathbf{c}||_1$=3.0

Integrand family		1	2	3	4	5	6
N		10000(0)	10000(0)	10000(0)	10000(0)	10000(0)	10000(0)
N_{train}		5000(0)	5000(0)	7000(0)	5000(0)	5000(0)	3000(0)
N_{crxn}		5000(0)	5000(0)	3000(0)	5000(0)	5000(0)	7000(0)
$\sigma_I/\lvert I\rvert$	VEG	$9(4)\times10^{-3}$	$5.97(8)\times10^{-4}$	$2.83(3)\times10^{-3}$	$5.89(5)\times10^{-4}$	$7.16(7)\times10^{-4}$	$4.2(3)\times10^{-3}$
	MLP	$1.8(7)\times10^{-4}$	$4.9(1)\times10^{-5}$	$4.5(2)\times10^{-4}$	$5.07(8)\times10^{-5}$	$1.45(5)\times10^{-4}$	$6.9(9)\times10^{-3}$
	GBDT	$9(4)\times10^{-3}$	$8.9(1)\times10^{-5}$	$6.76(8)\times10^{-3}$	$9.2(1)\times10^{-5}$	$4.61(5)\times10^{-4}$	$8.7(2)\times10^{-4}$
	GP	$8(3)\times10^{-5}$	$5(1)\times10^{-7}$	$2.45(4)\times10^{-5}$	$3.04(7)\times10^{-7}$	$4.9(1)\times10^{-4}$	$2.2(2)\times10^{-2}$
Gain	MLP	39.9(1.9)	12.32(27)	6.57(20)	11.70(19)	5.14(19)	0.722(41)
	GBDT	1.832(71)	6.70(14)	0.4227(74)	6.373(81)	1.561(28)	4.65(26)
	GP	118.1(1.1)	1679(111)	116.3(1.1)	1976(47)	1.488(40)	0.227(13)
$N_{>4\sigma}$	VEG	–	–	–	–	–	1

N≈10000, D=10, $||\mathbf{c}||_1$=8.0

Integrand family		1	2	3	4	5	6
N		10000(0)	10000(0)	10000(0)	10000(0)	10000(0)	10000(0)
N_{train}		5000(0)	5000(0)	7000(0)	5000(0)	5000(0)	3000(0)
N_{crxn}		5000(0)	5000(0)	3000(0)	5000(0)	5000(0)	7000(0)
$\sigma_I/\lvert I\rvert$	VEG	$2(1)\times10^{-2}$	$8.2(1)\times10^{-4}$	$7.06(9)\times10^{-3}$	$8.70(9)\times10^{-4}$	$9.21(9)\times10^{-4}$	$5.5(3)\times10^{-3}$
	MLP	$7(4)\times10^{-4}$	$2.59(3)\times10^{-4}$	$1.0(1)\times10^{-3}$	$3.31(5)\times10^{-4}$	$4.1(2)\times10^{-4}$	$7.6(8)\times10^{-3}$
	GBDT	$1(1)\times10^{-1}$	$5.30(8)\times10^{-4}$	$2.43(6)\times10^{-2}$	$6.37(7)\times10^{-4}$	$1.34(1)\times10^{-3}$	$2.44(5)\times10^{-3}$
	GP	$3(1)\times10^{-4}$	$4.5(8)\times10^{-5}$	$1.30(6)\times10^{-4}$	$2.39(6)\times10^{-6}$	$1.42(2)\times10^{-3}$	$2.9(3)\times10^{-2}$
Gain	MLP	42.69(95)	3.181(47)	7.67(36)	2.645(44)	2.39(10)	0.872(56)
	GBDT	1.100(26)	1.570(43)	0.2983(86)	1.371(19)	0.690(12)	2.198(93)
	GP	88.7(2.6)	35.6(4.0)	56.9(2.1)	372.1(9.0)	0.657(15)	0.236(17)
$N_{>4\sigma}$	VEG	–	–	–	–	–	2
	MLP	–	–	7	–	–	–
	GP	–	–	1	–	–	–

Table S6: Number of integrand evaluations, precision of the integral, precision gain, and $N_{>4\sigma}$ for $N \approx 10000$ and $D = 8$. Notations are the same as Table S1.
